# Probiotic supplements containing *Lactobacillus reuteri* does not affect the levels of matrix metalloproteinases and interferons in oral wound healing

**DOI:** 10.1186/s13104-018-3873-9

**Published:** 2018-10-25

**Authors:** Svante Twetman, Anne Marie Lynge Pedersen, Tulay Yucel-Lindberg

**Affiliations:** 1 Department of Odontology, Faculty of Health and Medical Sciences, University of Copenhagen, Nørre Allé 20, 2200 Copenhagen N, Denmark; 2 Division of Periodontology, Department of Dental Medicine, Karolinska Institutet, Box 4064, 141 04 Huddinge, Sweden

**Keywords:** Biopsy, Cytokines, Inflammation, Oral mucosa, Probiotics

## Abstract

**Objective:**

The use of beneficial bacteria may stimulate wound healing. We performed a randomized, placebo-controlled double-blind cross-over study comprising ten healthy volunteers. The aim was to investigate the impact of topical and systemic applications of probiotic lactobacilli (*Lactobacillus reuteri*) on the healing of standardized wounds (punch biopsies) in the oral mucosa. The expression of selected matrix metalloproteinases (MMP’S) and interferons (IFN’s) was analyzed with multiplex immunoassays in the wound exudate during the first healing week (day 2, 5 and 8).

**Results:**

All participants completed the study and in all cases, the healing after the punch biopsies was uneventful. The concentrations of MMP-1, MMP-2, MMP-3 decreased with time in both the test- and control group. The MMP levels were consistently lower during the probiotic intervention when compared with placebo but the differences were not statistically significant. Likewise, the concentrations if IFN-alpha2, IFN-beta and IFN-gamma decreased with time with no significant differences between the test and placebo interventions. Within the limitations of this pilot study, we were unable to demonstrate an influence of probiotic supplements containing *L. reuteri* on the concentrations of selected matrix metalloproteinases and interferons from mucosal wounds within 1 week after a standardized punch biopsy.

*Trial registration* ClinicalTrials.gov Identifier NCT03210779. Date of registration: July 7, 2017

**Electronic supplementary material:**

The online version of this article (10.1186/s13104-018-3873-9) contains supplementary material, which is available to authorized users.

## Introduction

Matrix metalloproteinases (MMP’s) is a family of proteolytic enzymes basically involved in tissue remodeling, wound healing and inflammation [1]. MMP’s are also suggested to play a role in biofilm-mediated oral diseases [2, 3] with elevated salivary concentrations in patients with active caries [4] and in patients with severe periodontitis [5]. The use of lactobacilli-derived probiotic bacteria has in recent years emerged as a novel strategy for the management of dysbiotic oral biofilms [6, 7] but little is known on its possible influence on the MMP’s in the oral cavity. Staab and co-workers [8] reported reduced levels of MMP-3 in gingival crevicular fluid (GCF) from healthy volunteers following intake of milk containing *L. casei*. Significantly decreased levels of MMP-8 have also been shown in patients with chronic periodontitis after intake of lozenges with *Lactobacillus reuteri* when compared with placebo [9]. On the other hand, Jäsberg et al. [10] demonstrated increased concentrations of MMP-9 in healthy volunteers after a 4-week intake of *B. lactis* and *L. rhamnosus* GG. Interferon (IFN) is a group of signaling proteins produced and released by host cells in response to the presence of pathogens or tissue damage. Previous research has indicated that probiotic treatment can decrease the expression of IFN-gamma from T-cells [11] and in saliva [12]. We have recently investigated *L. reuteri* in the context of oral wound healing and found elevated levels of ligand members of the tumor necrosis factor (TNF) superfamily, as well as Interleukin 8 in the wound exudate after a standardized punch biopsy [13]. In this communication, the levels of MMP-1, MMP-2, MMP-3, IFN-alpha2, IFN-beta and IFN-gamma are reported. The null hypothesis was that the concentrations would not differ from those obtained when exposed to placebo.

## Main text

### Materials and methods

The study group consisted of 10 healthy volunteers of both sexes (8 females, 2 males). The mean age was 29.5 years (range 21–66 years) and all were employees or students at the University Hospital. The inclusion criteria were non-compromised general and oral health and no regular intake of pharmaceuticals with the exception of contraceptives. Exclusion criteria were (a) smoking, (b) any severe allergy or bleeding disorder, and (c) a regular intake probiotic products. All participants had a normal appearing oral mucosa and no signs of dental caries or periodontal disease.

### Study design

The study had a randomized, placebo-controlled, double-blind cross-over design. The protocol was approved by The Research Ethics Committee of the Capital Region of Denmark (H-16025546) and the study was registered at Clintrials.gov (NCT03210779). During a 1-week run-in period, the participants were assigned to let an active or placebo lozenge slowly dissolve in their mouth, two times per day. After 8 days, a biopsy was taken under local anesthesia containing adrenalin with aid of a standardized punch (4 mm diameter) in the free buccal oral mucosa in the upper left or right premolar region. After compression with cotton gauges for 30 min, the created wound was left uncovered and no sutures were used. The subjects were thoroughly instructed to continue with their assigned lozenges for another 8 days. In addition, they were asked to topically apply one drop of oil (probiotic or placebo) directly on the wound, once daily for 8 days with aid of a plastic micro-brush. No food or nutritional restrictions were given, and all subjects were asked to maintain their normal oral hygiene routines during the course of the study. After a 4-week washout period, all the procedures were repeated a second time with either the test or placebo lozenges/oil.

### Intervention

The test lozenges (BioGaia ProDentis, BioGaia AB, Sweden) contained a mix of two probiotic strains, *L. reuteri* DSM 17938 and ATCC PTA 5289, with at least 5 × 10^8^ live bacteria of each strain per lozenge. The placebo lozenges had an identical composition, shape and taste, but without active bacteria. The test oil contained the same probiotic strains with a concentration of 2 × 10^8^ CFU/mL. The study products were provided in identical plastic containers, separated by a color code. The compliance was checked through a logbook that was filled in on a daily basis.

### Sample collection and biochemical assays

All subjects were recalled after 2, 5 and 8 days for check-ups. The wound healing was assessed with a 4-level clinical score as described in Additional file 2. Exudate from the wounds was collected by sterile circular standard filter papers (Sialostrip^®^, ProFlow™ Inc., Amenityville, MY, USA) that were applied on top of the wound for 20 s. The volume of liquid was determined with a Periotron^®^ 8000 (ProFlow). The filter papers were diluted in 150 µL of PBS (pH 7.4) with 0.05% Tween-20, vortexed for 30 s and stored at − 80 °C until further analysis. Commercial multiplex immunoassay kits (human inflammation panel, Bio-Rad, Hercules, CA, USA) were used to determine the MMP and interferon levels in exudate samples, handled according to manufacturer’s protocol. The analyzed enzymes (sensitivities in pg/mL, in parentheses) were MMP-1 (33.7), MMP-2 (39.7) and MMP-3 (28.5) and the cytokines were IFN-alpha2 (0.7), IFN-beta (2.0) and IFN-gamma (0.05). Total protein concentration, expressed as mg/mL, was analyzed using Bio-Rad DC protein assay (Bio-Rad, Solna, Sweden). The protein levels were measured in duplicates and the levels of MMPs and IFNs were analyzed in single within one analytical run, as previously described [13].

### Statistical methods

All data were processed with the IBM SPSS software (version 25.0, Chicago, USA). The differences between groups were analyzed by Wilcoxon unpaired test. *P*-values less than 0.05 were considered as statistically significant. The blinding was not unveiled until the statistical analyses were concluded.

### Results and discussion

All participants completed the study and in all cases, the healing after the punch biopsies was uneventful (Additional file 1). There were no statistically significant differences in the oral wound healing pattern between test and placebo (Additional file 2). The mean concentration of total protein and the selected MMP’s are shown in Table 1. The amount of wound exudate, as well as the concentration of total protein, decreased with time in a similar way in both groups. The MMP levels were consistently lower during the probiotic intervention when compared with placebo but the differences were not statistically significant. The mean concentrations of IFN-alpha2, IFN-beta and IFN-gamma are presented in Table 2. Also here, the measured levels decreased with time but with no significant differences between the test and placebo interventions.

**Table 1:** Amount of wound exudate and concentration of total protein and selected MMP’s in wound exudate collected 2, 5 and 8 days after a standardized punch biopsy, with and without exposure to probiotic supplements

Variables	Day 2	Day 5	Day 8
Wound exudate, µL			
Test	2.01 (0.54)	1.05 (0.46)	0.40 (0.29)
Placebo	2.00 (0.65)	0.99 (0.80)	0.34 (0.21)
Total protein, mg/mL			
Test	1.50 (0.22)	0.53 (0.18)	0.30 (0.13)
Placebo	1.84 (0.28)	0.52 (0.06)	0.22 (0.04)
MMP-1, pg/mL ×10^3^			
Test	3.51 (2.47)	3.05 (5.31)	0.86 (1.38)
Placebo	3.90 (3.84)	3.93 (4.77)	2.17 (5.85)
MMP-2, pg/mL ×10^3^			
Test	1.64 (0.75)	0.51 (0.56)	0.04 (0.06)
Placebo	1.57 (1.43)	0.76 (0.92)	0.19 (0.48)
MMP-3, pg/mL ×10^3^			
Test	1.06 (0.71)	0.67 (0.44)	0.16 (0.21)
Placebo	1.33 (1.02)	0.73 (0.62)	0.43 (1.20)

Values denote mean and standard deviation (SD)

**Table 2:** Concentration of selected interferons (pg/mL) in wound exudate collected 2, 5 and 8 days after a standardized punch biopsy, with and without exposure to probiotic supplements

Variables	Day 2	Day 5	Day 8
IFN-alpha2			
Test	23.5 (15.1)	13.7 (8.4)	4.2 (4.0)
Placebo	30.3 (7.7)	17.6 (19.0)	11.5 (25.3)
IFN-beta			
Test	20.2 (13.5)	14.0 (9.0)	4.1 (2.0)
Placebo	24.2 (6.7)	11.6 (7.6)	6.7 (9.2)
IFN-gamma			
Test	9.0 (6.3)	5.0 (4.3)	0.1 (0.2)
Placebo	11.4 (4.0)	4.2 (4.5)	1.9 (5.8)

Values denote mean and standard deviation (SD)

To our knowledge, this is the first study to investigate the possible impact of topical and systemic administration of probiotic supplements on a panel of inflammatory mediators related to oral wound healing. In particular, data on the concentration of MMP’s and IFN’s in the exudate have not previously been reported. We used a well-controlled cross-over design with run-in and wash-out periods of 1 and 4 weeks, respectively. Importantly, the participating subjects were exposed to the test or placebo interventions in a randomized order. Furthermore, the volunteers were healthy and well-fed and the compliance with the study protocol was excellent [13]. As we were unable to demonstrate any significant differences in the amounts of MMP’s and IFN’s between the two interventions, the null hypothesis was accepted. The results remained unaltered when the concentrations were related to the total amount of protein in the samples and to the output (volume/time) of the exudate.

The healing of oral mucosal wounds follows a well-described pathway of hemostasis, inflammation, proliferation and remodeling, regulated by a complex network of growth factors, cytokines and chemokines [14]. Interestingly, laboratory data indicate that IFN-gamma may regulate MMP’s involved in connective tissue turnover in the presence of TNF-alpha [15]. Previous clinical studies have suggested that beneficial bacteria may accelerate the healing of surgical wounds as well as burns and diabetic foot ulcers [16–19]. The precise mechanism of action is not fully known but a systemic influence on the immune response by balancing pro- and anti-inflammatory agents has been described [20, 21]. Based on previous research, we had hoped to unveil decreased amounts of MMP-3 [8] and IFN-gamma [12] in connection with the probiotic intervention, albeit the former analyses were carried out in gingival crevicular fluid and saliva, respectively. The fact that we were unable to disclose these events in the present pilot study does not exclude the possibility that beneficial bacteria can play a role in oral inflammation. We noticed a tendency towards lower levels of MMP’s after 8 days in the probiotic group but the limited number of participants and the large inter-individual variations recorded in the exudates made the study underpowered with respect to the biochemical variables. Another open question is whether or not there is a dose-relationship between exposure of oral probiotics and the expression of inflammatory mediators in saliva or gingival crevicular fluid. Thus, an expanded clinical trial with focus on recurrent or chronic oral wounds could be justified as such wounds are prevalent and a therapeutic challenge in an aging population.

### Conclusion

Within the limitations of this study, we were unable to demonstrate an influence of probiotic supplements containing *L. reuteri* on the concentrations of selected MMP’s and IFN’s in the wound exudate within 1 week after a standardized punch biopsy.

### Limitations

This was a pilot study and the sample size (α = 0.05 and β = 0.2) was estimated on clinical endpoints (healing score, pain and discomfort) rather than on any biochemical endpoint. The data were obtained from the healing of fresh oral wounds and the findings may not be generalized to chronic wounds. Compliance was not verified by primer-specific polymerase chain reaction (PCR) analyses of *L. reuteri* in the wound exudate.

## Additional files

**Additional file 1.** Oral wound healing 2, 5 and 8 days after a standardized punch biopsy.

**Additional file 2.** Distribution of clinical healing scores after a standardized punch biopsy in the oral mucosa with exposure to probiotic supplements or placebo.

## Abbreviations

CFU: colony forming units
GCF: gingival crevicular fluid
IFN: interferone
MMP: matrix metalloproteinase
TNF: tumour necrosis factor
