# Supplementary material for: Probiotic supplements containing Lactobacillus reuteri does not affect the levels of matrix metalloproteinases and interferons in oral wound healing
Source: BMC Res Notes. 2018 Oct 25;11:759. doi: 10.1186/s13104-018-3873-9 (PMC6203191; doi:10.1186/s13104-018-3873-9)

**Supplementary file 1:** Examples of clinical oral wound healing

1. Fresh punch biopsy (baseline) b) Healing after 2 days


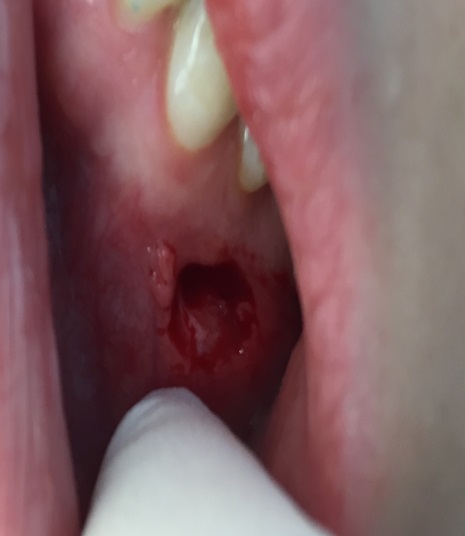

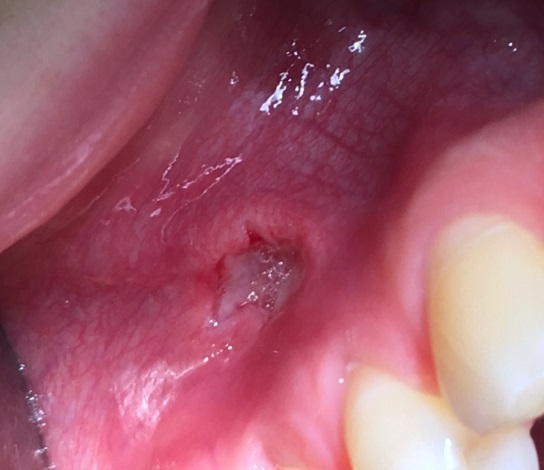


1. Healing after 5 days d) Healing after 8 days


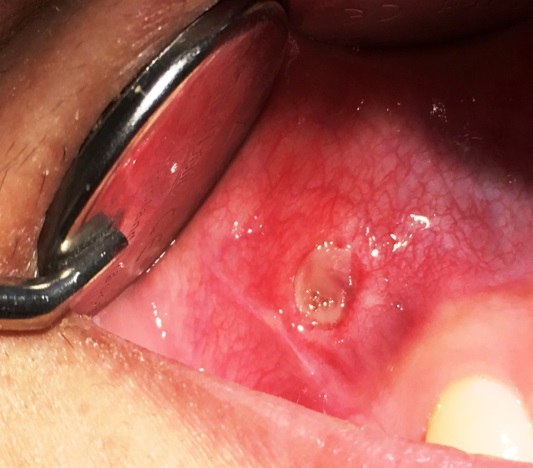

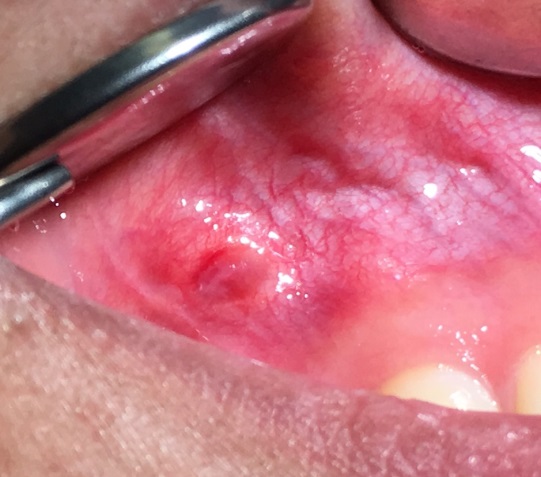

Supplement: Supplementary file 1 — Additional file 1. Oral wound healing 2, 5 and 8 days after a standardized punch biopsy. [file 13104_2018_3873_MOESM1_ESM.docx]
