# Supplementary material for: Probiotic supplements containing Lactobacillus reuteri does not affect the levels of matrix metalloproteinases and interferons in oral wound healing
Source: BMC Res Notes. 2018 Oct 25;11:759. doi: 10.1186/s13104-018-3873-9 (PMC6203191; doi:10.1186/s13104-018-3873-9)
Supplement: Supplementary file 2 — Additional file 2. Distribution of clinical healing scores after a standardized punch biopsy in the oral mucosa with exposure to probiotic supplements or placebo. [file 13104_2018_3873_MOESM2_ESM.docx]

**Supplementary file 2**

Distribution of clinical healing scores after a standardized punch biopsy in the oral mucosa with exposure to probiotic supplements containing *L. reuteri* (lozenges and topical oil application) or placebo. Values in the table denote number of subjects

__________________________________________________________________________

Wound healing Baseline Day 2 Day 5 Day 8

Test/placebo Test/placebo Test/placebo Test/placebo

__________________________________________________________________________

Score 3 10/10 0/0 0/0 0/0

Score 2 0/0 3/7 2/4 0/1

Score 1 0/0 7/3 8/6 8/5

Score 0 0/0 0/0 0/0 2/4

__________________________________________________________________________

**Score 3**: Fresh clean wound with initial hemorrhage

**Score 2**: >25% of lesion show red inflammatory response, edema with connective tissue exposed

**Score 1**: ≤25% of lesion show red inflammatory response, no/minor edema, fibrin-covered surface

**Score 0**: no signs of edema or inflammation, pink/pale lesion surface
